# MnTnHex-2-PyP^5+^ Displays Anticancer Properties and Enhances Cisplatin Effects in Non-Small Cell Lung Cancer Cells

**DOI:** 10.3390/antiox11112198

**Published:** 2022-11-07

**Authors:** Rita B. Soares, Rita Manguinhas, João G. Costa, Nuno Saraiva, Nuno Gil, Rafael Rosell, Sérgio P. Camões, Ines Batinic-Haberle, Ivan Spasojevic, Matilde Castro, Joana P. Miranda, Filipa Amaro, Joana Pinto, Ana S. Fernandes, Paula Guedes de Pinho, Nuno G. Oliveira

**Affiliations:** 1 Research Institute for Medicines (iMed.ULisboa), Faculty of Pharmacy, Universidade de Lisboa, Av. Professor Gama Pinto, 1649-003 Lisboa, Portugal; 2 Universidade Lusófona’s Research Center for Biosciences & Health Technologies (CBIOS), Campo Grande 376, 1749-024 Lisboa, Portugal; 3 Lung Unit, Champalimaud Clinical Centre, Champalimaud Foundation, Av. Brasília, 1400-038 Lisbon, Portugal; 4 Laboratory of Cellular and Molecular Biology, Institute for Health Science Research Germans Trias i Pujol (IGTP), Campus Can Ruti, Ctra de Can Ruti, Camí de les Escoles, s/n, 08916 Badalona, Barcelona, Spain; 5 Department of Radiation Oncology, Duke University School of Medicine, Durham, NC 27710, USA; 6 Department of Medicine, Duke University School of Medicine and PK/PD Core Laboratory, Duke Cancer Institute, Duke University School of Medicine, Durham, NC 27710, USA; 7 Associate Laboratory i4HB-Institute for Health and Bioeconomy, Department of Biological Sciences, Laboratory of Toxicology, Faculty of Pharmacy, University of Porto, 4050-313 Porto, Portugal; 8 UCIBIO, REQUIMTE, Laboratory of Toxicology, Faculty of Pharmacy, University of Porto, 4050-313 Porto, Portugal

**Keywords:** non-small cell lung cancer, SOD mimic, MnTnHex-2-PyP^5+^, metabolomics, cytotoxicity, cisplatin, antioxidant enzymes, migration

## Abstract

The manganese(III) porphyrin MnTnHex-2-PyP^5+^ (MnTnHex) is a potent superoxide dismutase mimic and modulator of redox-based transcriptional activity that has been studied in the context of different human disease models, including cancer. Nevertheless, for lung cancer, hardly any information is available. Thus, the present work aims to fill this gap and reports the effects of MnTnHex in non-small cell lung cancer (NSCLC) cells, more specifically, A549 and H1975 cells, in vitro. Both cell lines were initially characterized in terms of innate levels of catalase, glutathione peroxidase 1, and peroxiredoxins 1 and 2. To assess the effect of MnTnHex in NSCLC, alone or in combination with cisplatin, endpoints related to the cell viability, cell cycle distribution, cell motility, and characterization of the volatile carbonyl compounds (VCCs) generated in the extracellular medium (i.e., exometabolome) were addressed. The results show that MnTnHex as a single drug markedly reduced the viability of both NSCLC cell lines, with some IC_50_ values reaching sub-micromolar levels. This redox-active drug also altered the cell cycle distribution, induced cell death, and increased the cytotoxicity pattern of cisplatin. MnTnHex also reduced collective cell migration. Finally, the metabolomics study revealed an increase in the levels of a few VCCs associated with oxidative stress in MnTnHex-treated cells. Altogether these results suggest the therapeutic potential of MnTnHex to be further explored, either alone or in combination therapy with cisplatin, in NSCLC.

## 1. Introduction

Globally, lung cancer (LC) continues to be the leading cause of cancer-related deaths for both genders [1,2]. Non-small cell lung cancer (NSCLC), one of the major subtypes of LC, is responsible for up to 85% of all LC cases, of which almost half are adenocarcinomas [3,4]. Most NSCLC cases are detected in advanced stages when metastases have already developed, which decreases the survival rate to very low % values [5,6,7]. In these advanced stages (IIIB-IV), a multidisciplinary treatment is the most common practice. Platinum-based chemotherapy alone or combined with radiotherapy has been used in most treatment protocols [8], cisplatin being one of the most common platinum analogs used. The mode of action of cisplatin consists of binding to nuclear DNA thereby creating DNA adducts. Particularly, cisplatin reacts with the N-7 of purines, generating intra- and inter-strand cross-links [9] and, consequently, blocks replication and transcription, which results in cell death by necrosis or apoptosis [10]. However, this mode of action leads to an indiscriminate attack on all rapidly dividing cells, which results in well-established adverse side effects. The most common side effects include nephrotoxicity, ototoxicity, neurotoxicity, and vomiting [10,11,12]. Moreover, the development of resistance of cancer cells to cisplatin and its analogs is a major issue [12].

Oxidative stress is present in several pathologies, including cancer [13], and there have been several reports addressing this topic. Higher concentrations of reactive oxygen species (ROS) are commonly found in cancer cells when compared with normal cells [14,15,16]. More specifically, these cells have a generally lower capability to remove hydrogen peroxide (H_2_O_2_) [17]. The ability to detoxify H_2_O_2_ depends largely on the levels of catalase (CAT), glutathione peroxidase (GPx), and peroxiredoxins (PRDX) [18]. These enzymes are either present at lower levels in malignant cells or are inactivated. Such a situation enables cancer cells to employ ROS for their own benefit, i.e., survival and proliferation. Yet, the balance is delicate, and if the ROS are pushed to very high levels, they would become cytotoxic to cancer cells and would force them to undergo apoptosis/necrosis [19]. Thus, different approaches to redox modulation that further increase the concentrations of ROS, particularly H_2_O_2_, are advantageous in a therapeutic context. This concept has been termed redox-directed cancer therapy [20] and is a promising strategy to enhance the efficacy of chemotherapy in suppressing the growth of these cells.

Using native superoxide dismutases (SOD) was formerly suggested as a possible therapeutic strategy [21,22]. These enzymes are responsible for the dismutation of superoxide anion (O_2_^•−^), thus accumulating H_2_O_2_. The clinical use of native SOD has its disadvantages, such as elevated manufacturing costs, low permeability, high immunogenicity, and short half-life [23]. To overcome these disadvantages, SOD mimics (SODm) were initially created. These are synthetic compounds with the ability to mimic the properties of native SOD enzymes [24]. SODm are capable of disproportionating O_2_^•−^ and also possess scavenging properties towards other reactive species, such as ONOO^−^ and NO_2_ [14]. These compounds may also increase the amount of H_2_O_2_ present in the cells either via O_2_^•−^ dismutation or cycling with cellular reductants [25], and most so in cancer cells where the H_2_O_2_-removing systems are deficient. This could push H_2_O_2_ concentration to the toxicity limit, reducing proliferation and increasing the efficiency of chemotherapy and radiotherapy [14,26,27]. Apart from these benefits, SODm can also protect healthy cells against the adverse effects of chemo/radiotherapy since, in general, as abovementioned, non-malignant cells display higher basal levels of antioxidants, therefore counteracting the increase in H_2_O_2_ levels. In this sense, the SODm have a dual role [14,23], being currently considered modulators of cellular transcriptional activity in both cancer and healthy cells [28]. 

**Figure 1:**
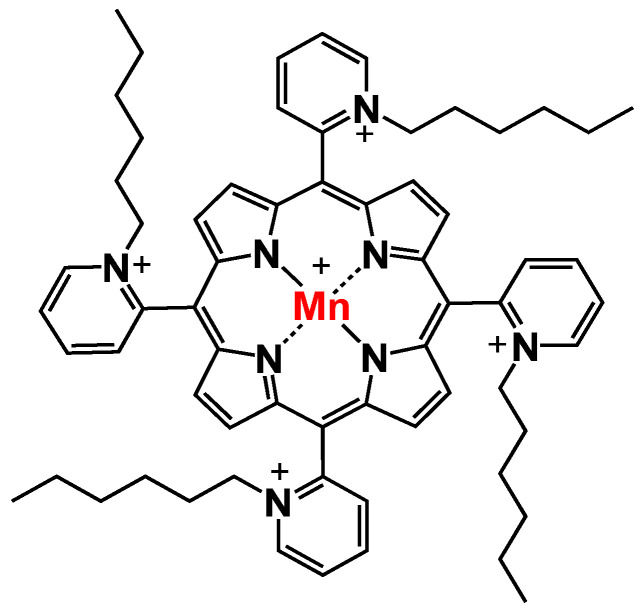
Chemical structure of MnTnHex-2-PyP^5+^ (chemical name: Mn(III)*meso*-tetrakis(*N*-n-hexylpyridinium-2-yl)porphyrin), designated as MnTnHex throughout the text for simplicity.

Manganese porphyrins (MnPs) are one of the most promising classes of SODm. These porphyrins are highly stable and can penetrate cellular membranes due to their low molecular weight [28]. Also, they have been shown to inhibit transcription factors such as HIF-1α, NF-κB, and AP-1 but activate Nrf2 [25,28]. One of the most promising MnPs is *ortho* Mn(III) *meso*-tetrakis(*N*-n-Hexylpyridinium-2-yl)porphyrin (MnTnHex-2-PyP^5+^, MnTnHex, Figure 1). This redox-active compound is the most lipophilic MnP, having high bioavailability and distribution while at the same time displaying low toxicity in healthy cells and tissues [25]. MnTnHex has been studied in different types of cancer, e.g., breast, renal cancer, and glioblastoma [24,29,30,31]. It was also found to reduce the viability and migration of renal metastatic cancer cells [29]. In addition, when combined with doxorubicin (dox), MnTnHex reduced the migration of breast cancer cells [24]. In mice, MnTnHex was able to significantly delay brain tumor growth by 3 to 34 days, depending on the xenograft [31]. 

In a comparative study regarding the radioprotective effect of several antioxidant manganese compounds in ataxia-telangiectasia lymphoblastoid cells and wildtype (WT) cells derived from healthy individuals, MnTnHex was the most effective in protecting WT cells from radiation-induced apoptosis and DNA damage at nM levels [32]. This compound was also studied in V79 lung fibroblasts and did not exhibit cytotoxicity [33]. Other studies have addressed the radioprotective effect of MnTnHex in the lungs. Cline et al. [34] demonstrated that MnTnHex could delay radiation-induced lung lesions in non-human primates. Also, at the concentration of 0.05 mg/kg, MnTnHex did not show any evidence of lung cytotoxicity. 

Regarding lung cancer cells, there is, however, a lack of information considering the cytotoxic effects of MnPs. The present study intends to fill this gap. In this report, the impact of MnTnHex, either as a single drug or combined with cisplatin, was assessed in NSCLC cell lines (A549 and H1975). Complementary endpoints related to cell viability, migration, and cell cycle distribution, were studied for the first time. Furthermore, this report provides molecular clues regarding the impact of MnTnHex and cisplatin on the extracellular metabolic profile of the two NSCLC cell lines using metabolomics as an innovative tool. This tool measures the levels of low molecular weight molecules participating in cellular biochemical processes, providing novel insights into the cellular metabolic response to MnTnHex and cisplatin.

## 2. Materials and Methods

### 2.1. Chemicals

RPMI-1640 with L-glutamine was purchased from Biowest (Nuaillé, France). MnTnHex was synthesized and characterized at Duke University School of Medicine, according to Batinic-Haberle et al. [35]. Cisplatin, penicillin-streptomycin solution (10,000 units/mL of penicillin; 10 mg/mL of streptomycin), crystal violet, sodium bicarbonate, and O-(2,3,4,5,6-pentafluorobenzyl) hydroxylamine (PFBHA, ≥99%) were obtained from Sigma-Aldrich (Madrid, Spain). Ethanol absolute and acetic acid were purchased from Merck (Darmstadt, Germany). Sodium pyruvate was purchased from Lonza (Basel, Switzerland) and trypsin (0.25%), and fetal bovine serum (FBS) from Gibco (Eugene, OR, USA). HEPES and D-Glucose were obtained from AppliChem (Darmstadt, Germany). CellTiter 96^®^ Aqueous MTS (3-(4,5-Dimethylthiazol-2-yl)-5-(3-carboxylmethoxyphenyl)-2-(4-sulfophenyl)-2H-tetrazolium) was acquired from Promega (Madison, WI, USA).

The stock solution of MnTnHex and its dilutions were prepared in Milli-Q water. Cisplatin was dissolved in saline solution (0.9% NaCl), and its aliquoted solutions were stored at −20 °C. In all cell-based assays, controls were also included, in which cells were exposed to either saline solution or Milli-Q water.

### 2.2. Cell Culture

The human NSCLC cell lines A549 and H1975 were obtained from the American Type Culture Collection (ATCC, Manassas, VA, USA). Both cell lines were cultured in monolayer in RPMI-1640 medium with L-glutamine supplemented with 10 mM HEPES, 2.5 g/L D-glucose, 1 mM sodium pyruvate, 1.5 g/L sodium bicarbonate, 10% FBS, and 1% Pen/Strep (complete cell culture medium) and were maintained at 37 °C under a humidified atmosphere containing 5% CO_2_ in the air.

### 2.3. Gene Expression

The gene expression of the main antioxidant enzymes responsible for detoxifying H_2_O_2_ (*CAT, GPX, PRDX*) was assessed using quantitative reverse transcription PCR (RT-qPCR), following a previously described protocol [36]. RNA isolation was performed using TRIzol™ Reagent (Invitrogen, Massachusetts, USA) and quantified for cDNA synthesis. cDNA synthesis was obtained from 0.5 μg of RNA using a commercially available kit (NZYTech, Lisbon, Portugal). The qPCR was performed using PowerUp™ SYBR^®^ Green Master Mix (Applied Biosystems^®^/Life Technologies, Austin, TX, USA), according to the manufacturer’s instructions. A final reaction volume of 15 μL, with 1 μL of template cDNA and 0.1 μM of forward and reverse primers, was used, and the reaction was conducted in the QuantStudio™ 7 Flex Real-Time PCR System (Applied Biosystems™, Massachusetts, EUA). The forward and reverse primers used are present in Table 1. The amplification of cDNA comprised of denaturation at 95 °C for 10 min, 40 cycles of denaturation at 95 °C for 15 s, annealing at 60 °C for 1 min, and extension at 72 °C for 30 s. As a quality and specificity measurement, a dissociation stage was added to determine the melting temperature in all runs. The relative expression of target genes in each cell line was calculated using the expression 2^−ΔCt^, where ΔCt = Average Ct (gene of interest)—Average Ct (β-ACTIN) [37] and normalized them to the reference gene β-ACTIN.

### 2.4. Crystal Violet (CV) Staining Assay

The cytotoxic effects of MnTnHex alone in both NSCLC cell lines were first evaluated by the CV staining assay, following a previously described protocol [42]. Briefly, cells were seeded at 3 × 10^3^ cells/well in 200 µL of a complete medium in 96-well plates and incubated for 24 h at 37 °C under a 5% CO_2_ atmosphere. After the 24 h period, the culture medium was renewed, and the cells were incubated with a range of MnTnHex (0.5−25 µM) concentrations for 72 h. Cells were also incubated with 50 µM of cisplatin as a positive control. After incubation, cells were washed with PBS to remove non-adherent cells (non-viable cells). The adherent cells were fixed with ice-cold 96% ethanol for 15 min and stained with 0.1% CV for 5 min. After washing the microplate with tap water, the stained cells were resuspended in 200 µL of 96% ethanol/1% acetic acid. The absorbance was measured at 595 nm (OD595) using a SPECTROstar OMEGA microplate reader (BMG Labtech, Offenburg, Germany). Absorbance values presented by control cells corresponded to 100% of cell viability. Three independent experiments were performed, and six replicates were used for each condition in each independent experiment. The half-maximal inhibitory concentration (IC_50_) was calculated based on the concentration-response curve using GraphPad Prism^®^ 7.0 (GraphPad Software, Inc., La Jolla, CA, USA). Image acquisition was performed using a Motic AE2000 Inverted Phase Contrast Microscope and a 40 × objective. 

### 2.5. MTS Reduction Assay

The MTS reduction assay was performed as a complementary cell viability assay by applying the same conditions as in the CV assay and following a previously described protocol [43]. Briefly, after treatment with MnTnHex, cells were washed with PBS, followed by the addition of 100 µL of new complete medium and 20 µL of MTS substrate prepared from the CellTiter 96^®^ Aqueous MTS, according to the manufacturer’s instructions. Cells were incubated for 2 h, and the results were measured as absorbance at 490 nm and 690 nm (reference wavelength) using a SPECTROstar OMEGA microplate reader. Absorbance values presented by control cells corresponded to 100% of cell viability. Four independent experiments were performed, and three replicates were used for each condition in each independent experiment. The IC_50_ was also calculated based on the concentration-response curve using GraphPad Prism^®^ 7.0 (GraphPad Software, Inc., La Jolla, CA, USA).

### 2.6. Combinatory Assays with MnTnHex and Cisplatin 

The cytotoxic profile of MnTnHex combined with cisplatin in A549 and H1975 cells was evaluated by the CV staining assay, following a similar protocol as previously described. After the initial 24 h incubation period, the culture medium was changed, and cells were exposed to both compounds simultaneously for 72 h. Two concentrations of MnTnHex were used for both cell lines (0.5 and 1 µM). Cisplatin concentrations were 1 and 2 µM for A549 cells and 1 and 5 µM for H1975 cells. Four independent experiments were performed, and six replicates were used for each condition in each independent experiment. 

### 2.7. Cell Cycle Analysis

To assess the effect of the compounds on cell cycle distribution, cell DNA content was assessed by flow cytometry, following a previously described protocol [44]. Briefly, A549 and H1975 cells were seeded in 6-well plates at approximately 5 × 10^4^ and 6 × 10^4^ cells/well, respectively, and incubated for 24 h. After this period, the culture medium was changed, and cells were exposed to MnTnHex (0.5 and 1 µM) and cisplatin (1 µM), both alone or in combination, for 72 h. The medium was collected, and the cells were detached using a 5 mM EDTA solution in PBS at 37 °C. Next, they were washed with cold PBS and fixed with ice-cold 80% ethanol. Cells were stained with propidium iodide (10 µg/mL) and treated simultaneously with RNase A (20 µg/L) for 15 min and were then analyzed using a Cytek Aurora flow cytometer (Cytek Biosciences, Fremont, CA, USA). Data acquisition was performed using Cytek SpectroFlo software (Cytek Biosciences, Fremont, CA, USA) and was analyzed with FlowJo^®^ (Tree Star Inc., San Carlos, CA, USA). Three to four independent experiments were performed.

### 2.8. Migration 

#### 2.8.1. Selection of MnTnHex and Cisplatin Concentrations for the Migration Assay

To select the suitable non-toxic concentrations for the migration assay, the MTS reduction assay was performed in A549 and H1975 cell lines, as previously described [43]. A low serum content medium was used to minimize cell proliferation. Approximately 8 × 10^3^ cells/well were seeded in 96-well plates in a complete culture medium for 24 h. After this period, the complete medium was replaced by a new cell culture medium containing 2% FBS. Cells were incubated with a range of low concentrations of MnTnHex (0.25−5 µM) or cisplatin (0.25−1 µM) for 32h in both cell lines. The MTS assay was performed, and non-toxic concentrations of both compounds were selected (Figure S1). Three independent experiments were performed, and three replicates were used for each condition in each independent experiment.

#### 2.8.2. In Vitro Wound-Healing Assay

The in vitro wound-healing assay was performed to determine the collective cell migration ability in both NSCLC cells. This assay was performed according to a previously described protocol [26,44]. Briefly, A549 and H1975 cells were seeded in 24-well plates at approximately 8 × 10^4^ and 6.58 × 10^4^ cells/well, respectively, and incubated for 24 h in a complete cell culture medium. After this period, the culture medium was removed, and the injury in the cell monolayer was performed using a 200 µL sterile pipette tip, resulting in a scratch of approximately 0.6−0.8 mm wide. Cells were then washed with PBS to eliminate cellular debris and incubated with a culture medium containing 2% FBS and the desired compounds. A549 cells migrated in the presence of MnTnHex (5 µM) and/or cisplatin (0.5 µM). H1975 cells migrated in the presence of MnTnHex (0.5 µM) and/or cisplatin (1 µM). Both cell lines migrated for 32 h. Image acquisition was performed using a Motic AE2000 Inverted Phase Contrast Microscope and a 40× objective. Scratch width was measured using Motic Images plus v2.0 software (Motic, Barcelona, Spain) at 0, 8, 24, and 32 h for A549 cells and at 0, 20, 24, and 32 h for H1975 cells after the injury. The initial time-point was considered 0% of wound closure, and the percentage of cell migration was calculated based on the initial distance. At each time point, two pictures of each scratch were taken for each condition. Three different measures were made for each picture. Three to four independent experiments were performed.

### 2.9. Metabolomics

#### 2.9.1. Cell Culture and Collection of the Extracellular Medium

To assess the exometabolome (extracellular metabolites present in culture medium) of cells when exposed to MnTnHex and/or cisplatin, sample collection and preparation for analysis were performed according to a previously described protocol [45]. Briefly, A549 and H975 cells were seeded in 6-well plates at a density of approximately 8 × 10^4^ and 9 × 10^4^ cells/well, respectively, and incubated for 24 h in a complete cell culture medium. After this incubation period, the medium was renewed, and cells were exposed to the compounds for 72 h, using the same concentrations as in the cell cycle assay. Six to seven independent experiments were performed. The extracellular media from each condition and blanks (medium without cells) were collected and centrifuged (1200× *g*, 5 min, 4 °C). Then, the supernatant was collected and immediately stored at −80 °C until analysis. A pool of the extracellular media of all samples and blanks was also prepared to be used as quality control (QCs) to check the analytical reproducibility of the experiment.

#### 2.9.2. GC-MS Analysis: Sample Preparation and Analytical Conditions

The GC-MS analysis was focused on the profile of volatile carbonyl compounds (VCCs), which include several aldehydes and ketones that may be produced as a result of oxidative stress. The preparation of all samples and VCCs extraction was performed according to previously described protocols based on headspace solid-phase microextraction (HS-SPME) [45,46]. Briefly, frozen samples were thawed slowly at room temperature. The analysis of VCCs was performed by placing 1.5 mL of culture medium in a 10 mL glass vial together with 35 μL of the derivatizing agent (PFBHA, 40 g/L). The samples were incubated for 6 min at 62 °C, followed by an extraction step of VCCs from the sample headspace using a polydimethylsiloxane/divinylbenzene (PDMS/DVB) fiber at the same temperature for 51 min, under continuous stirring (250 rpm). To ensure and evaluate the reproducibility of the analyses, all samples were randomly injected, and the QCs were injected under the same conditions on every 7 samples. The VCCs adsorbed to the fiber were then analyzed in a 436-GC system (Bruker Daltonics, Fremont, CA) coupled with an EVOQ Triple Quadrupole (TQ) mass detector using a Bruker MS workstation software (version 8.2, Bruker Daltonics, Bremen, Germany). A fused silica capillary column Rxi-5Sil MS (30 m × 0.25 mm × 0.25 μm; RESTEK Corporation, U.S., Bellefonte, Pennsylvania) was used, and helium C-60 (Gasin, Portugal) was chosen as the carrier gas (flow rate 1 mL/min). The oven temperature was settled at 40 °C for 1 min, rising to 250 °C (rate 5 °C/min), held for 5 min, followed by increasing to 300 °C (rate 20 °C/min). The MS detector was operated in the electron impact mode (70 eV) at 270 °C. The temperature of the transfer line was 260 °C, and the manifold was 40 °C. Data acquisition was performed in full scan mode within an *m/z* mass range between 35−600 *m/z* with a scan time of 250 ms. 

#### 2.9.3. Compound Identification and GC-MS Data Pre-Processing

To identify the VCCs detected in the GC-MS chromatogram, a comparison between the MS fragmentation and the mass spectra present in the National Institute of Standards and Technology (NIST 14) database was performed, as well as a comparison with the experimental Kovats retention index (RI) and the ones present in the literature. When possible, the identification of the VCCs was confirmed by comparing the retention time (RT), and MS spectra of the samples with the commercially available standard compounds analyzed under the same conditions. All VCCs identified in culture media are indicated in Table S1, as well as in a representative chromatogram (Figure S2). The GC-MS chromatograms were converted to netCDF and pre-processed in MZmine−2.53 [47]. The pre-processing steps included crop filtering (*m/z* range 50−500, RT range 14.50−49.00 min), peak detection (noise level 5 × 10^4^), chromatogram builder (minimum 5 scans, intensity threshold 1 × 10^5^, minimum highest intensity 5 × 10^4^), deconvolution (minimum peak height 1 × 10^5^, baseline level 5 × 10^4^, peak duration 0.02−0.5 min, minimum data points 5), and alignment (*m/z* tolerance 0.1, RT tolerance 0.2). The pre-processed matrix was filtered by excluding all *m/z*-RT pairs with a relative standard deviation (RSD) higher than 30% considering the QCs (*n* = 12), followed by normalization of the area of each *m/z*-RT pair by the total area of all *m/z*-RT pairs with RSD < 30%.

#### 2.9.4. Statistical Analyses of the GC-MS Data

Principal component analysis (PCA) and partial least squares discriminant analysis (PLS-DA) were used to provide visual representations of the similarity and variability among the pre-processed GC-MS data for QCs and each condition under study. The identification of VCCs differing between the classes under study was performed using Volcano plots which represented the *p*-values from a non-parametric test (Mann-Whitney test) and the fold-change (FC) values between two groups under study (cells exposed to MnTnHex and cisplatin alone or combined vs. non-exposed cells) for all *m/z*-RT pairs. The PCA, PLS-DA, and Volcano plots were performed in the MetaboAnalyst 5.0 [48]. Finally, the levels of the VCCs selected in the Volcano plots were compared among the groups under study by Kruskal-Wallis ANOVA and represented in boxplots using GraphPad Prism (version 9, San Diego, CA, USA). Statistical significance was considered for *p*-value < 0.05 (95% confidence interval).

## 3. Results

### 3.1. A549 and H1975 Cell Lines Express Low Catalase Levels

**Figure 2:**
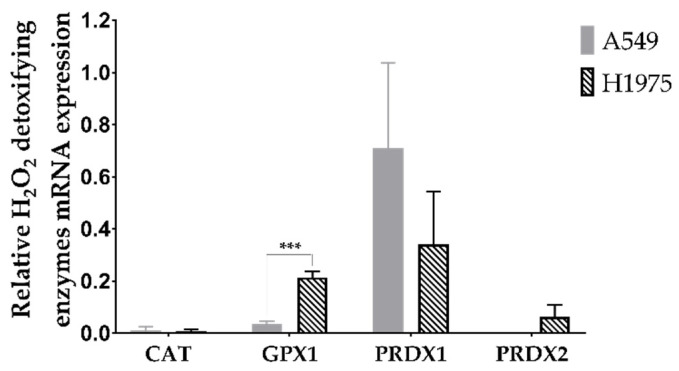
Gene expression analyses of the main H_2_O_2_ detoxifying enzymes. Values represent mean ± SD (*n* = 3) and are normalized to the reference gene β-ACTIN. *** *p* < 0.001 (Student’s *t*-test).

To determine the expression of the main antioxidant enzymes responsible for detoxifying H_2_O_2_, a qRT-PCR was conducted (Figure 2). According to this analysis, the gene expression levels for CAT were quite low in both cell lines. CAT is a pivotal cytosolic antioxidant enzyme responsible for dismuting H_2_O_2_ into H_2_O and O_2_. Regarding GPX1 (another cytosolic enzyme and major intracellular antioxidant enzyme), low levels were present, particularly in A549 cells. PRDX isoforms 1 and 2 are mainly localized in cytosol. Amongst the PRDX family, isoform 1 possesses the widest cellular distribution and also displays the highest abundance in various tissues. It is also the isoform most resistant to peroxidation damage within a wide range of H_2_O_2_ [49].

Importantly, we found that PRDX1 has high mRNA expression levels in both cell lines. The expression of this gene was more than 2-fold higher in A549 than in H1975 cells, albeit this difference was not significant. PRDX2 (an isoform more sensitive to peroxidation than PRDX1 [50]) was present in H1975 cells at low levels, these levels being even lower in A549 cells under our experimental conditions. The A549 cell line also has a lower level of GPX1 than the H1975 cells, this difference being significant (*p* < 0.001).

### 3.2. MnTnHex as a Single Drug Displays a Marked Cytotoxic Effect in NSCLC Cells

**Figure 3:**
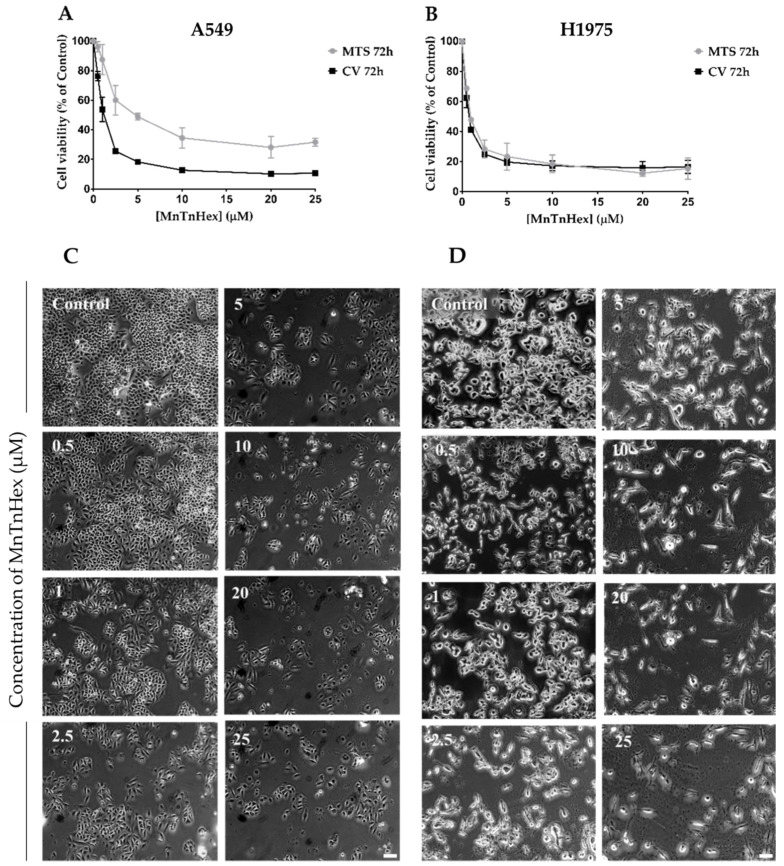
Cytotoxic effects of MnTnHex (0.5–25 μM) in A549 and H1975 cells. The decrease in cell viability upon exposure to MnTnHex, for 72 h, was assessed by CV and MTS assays in (**A**) A549 and (**B**) H1975 cells. Values represent mean ± SD (*n* = 3–4) and are expressed as percentages of the vehicle-treated control cells. (**C**) A549 cell morphology upon treatment with MnTnHex. (**D**) H1975 cell morphology upon treatment with MnTnHex. Scale bar = 100 μm.

To establish the cytotoxic profile of MnTnHex in A549 and H1975 cell lines, two different methodologies were used, the CV staining assay and the MTS reduction assay. In both assays and for both NSCLC cell lines, MnTnHex exhibited a concentration-dependent decrease in cell viability (0.5−25 µM) (Figure 3). The IC_50_ values for the CV assay were 0.9 µM and 0.7 µM for A549 and H1975 cells, respectively. Moreover, the IC_50_ values for the MTS assay were 2.1 µM in A549 cells and 1.0 µM for H1975 cells. From these results, it is clear that MnTnHex exhibited marked cytotoxic effects in both NSCLC cells. However, in H1975 cells, this compound displayed IC_50_ values lower than the IC_50_ of A549 cells, thus being more cytotoxic (Table S2). The morphology of cells was also analyzed, suggesting that A549 cells roughly maintain their morphology upon increasing MnTnHex concentrations (Figure 3C). However, in the case of H1975 cells, the morphology appears to be altered for higher MnTnHex concentrations. Indeed, the cells appear to be more elongated than control cells (Figure 3D). Finally, considering these results, MnTnHex concentrations of 0.5 and 1 µM were selected for combinatory assays in both cell lines since these concentrations induced distinct levels of cytotoxicity.

### 3.3. MnTnHex Enhances the Cytotoxicity of Cisplatin

**Figure 4:**
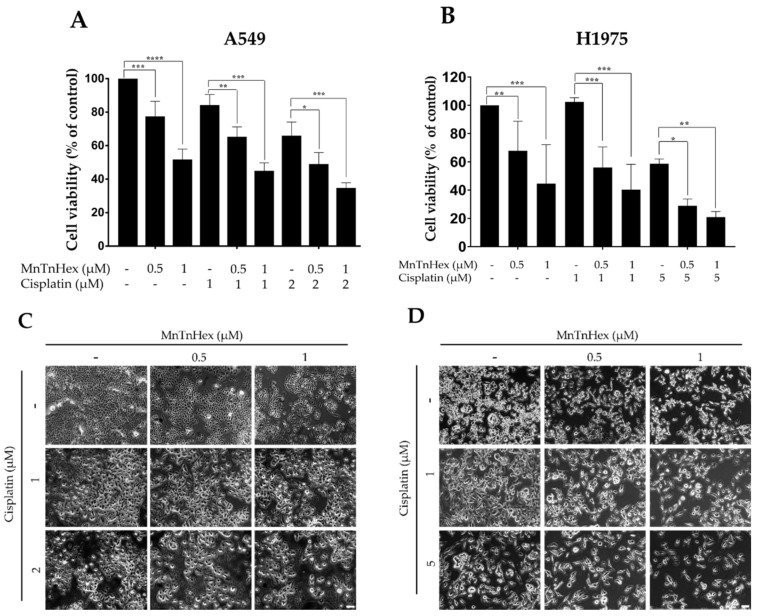
Cytotoxic effect of MnTnHex combined with cisplatin in A549 and H1975 cells. Cells were simultaneously treated with (**A**) MnTnHex (0.5 and 1 μM) and cisplatin (1 and 2 μM) in A549 cells and (**B**) MnTnHex (0.5 and 1 μM) and cisplatin (1 and 5 μM) in H1975 cells, for 72 h. This effect was evaluated using a CV assay. (**C**) A549 cell morphology upon treatment with MnTnHex and/or cisplatin. (**D**) H1975 cell morphology upon treatment with MnTnHex and/or cisplatin. Values represent mean ± SD (n = 4) and are expressed as percentages relative to control cells. * *p* < 0.05, ** *p* < 0.01, *** *p* < 0.001 and **** *p* < 0.0001 (one-way ANOVA with Tukey’s multiple comparisons test) when compared with control and cisplatin-treated cells. Scale bar = 100 μm.

To assess the effect of MnTnHex combined with cisplatin, NSCLC cells were exposed to both compounds simultaneously, and the effects were evaluated using the CV assay. For all combinations tested, a significant decrease in cell viability when compared to the cisplatin-treated cells was observed (Figure 4). In A549 cells, the cytotoxicity of cisplatin alone was slight to moderate at 1 and 2 µM, respectively, which was increased when combined with MnTnHex. In absolute percentage values, the decrease in cell viability observed for 1 µM of cisplatin, when combined with 0.5 and 1 µM of MnTnHex, was 18.8% (*p* < 0.01) and 39.2% (*p* < 0.001), respectively, and for 2 µM of cisplatin, when combined with the same MnTnHex concentrations, 17.0% (*p* < 0.05) and 31.2% (*p* < 0.001) (Figure 4A). 

In H1975 cells, the lowest cisplatin concentration used (1 µM) did not show any cytotoxicity, while the higher concentration (5 µM) displayed clear cytotoxic effects. The concomitant treatment of cisplatin with MnTnHex significantly decreased cell viability when compared with cisplatin alone. In absolute percentages, the combination of 1 µM of cisplatin with 0.5 and 1 µM of MnTnHex reduced cell viability by 46.4% (*p* < 0.001) and 62.1% (*p* < 0.001), respectively. Combining 5 µM cisplatin with the same MnP concentrations led to a reduction in cell viability of 29.7% (*p* < 0.05) and 37.8% (*p* < 0.01), respectively (Figure 4B). The morphology of A549 and H1975 cells upon treatment with MnTnHex and cisplatin are depicted in Figure 4C and Figure 4D, respectively. Overall, by combining MnTnHex with cisplatin, the cytotoxicity of this chemotherapeutic drug was significantly enhanced in NSCLC in vitro, this combination being more efficient in H1975 cells.

### 3.4. MnTnHex Enhances Cisplatin-Induced Cell Death

**Figure 5:**
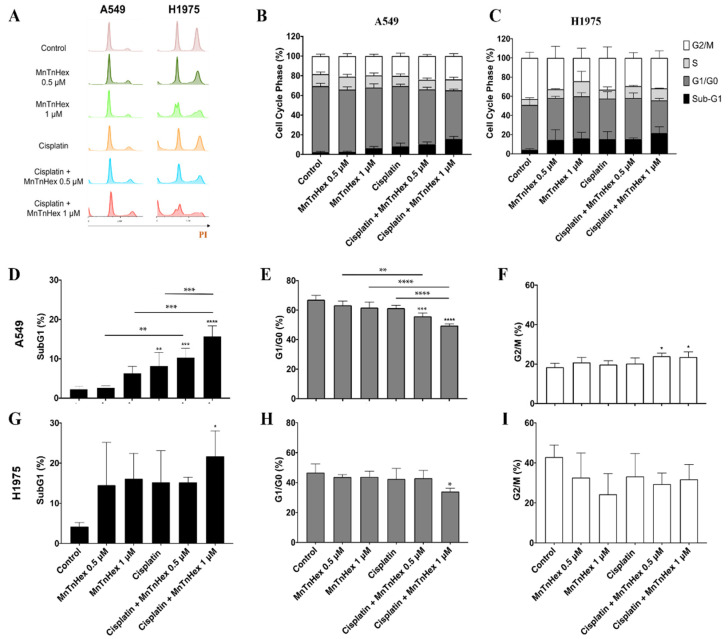
Effect of MnTnHex combined with cisplatin in A549 and H1975 cells on cell cycle distribution. Cells were treated with the indicated concentrations of MnTnHex and cisplatin (1 µM) for 72 h and DNA content was assessed on fixed cells by flow cytometry. (**A**) Representative flow cytometry histograms of PI-stained cells. Summary data of cell cycle populations for (**B**) A549 and (**C**) H1975 cells. Individual populations for (**D**–**F**) A549 and (**G**–**I**) H1975 cells. Values represent mean ± SD (*n* = 3–4) and are expressed as percentages of total cells. * *p* < 0.05, ** *p* < 0.01, *** *p* < 0.001 and **** *p* < 0.0001 (one-way ANOVA with Tukey’s multiple comparisons test) when compared with control cells, or between any two conditions as indicated in the figure by connecting lines.

The induction of cell death and the cell cycle progression was analyzed by flow cytometry (Figure 5). The cellular DNA content assessment showed that MnTnHex per se increased the percentage of the sub-G1 population in both cell lines at 1 µM. This was also observed at 0.5 µM but only for H1975 cells. Importantly, a strong increase in the percentage of the sub-G1 population was found when cisplatin was added in combination with MnTnHex (Figure 5D,G) in both cell lines. In terms of magnitude, the combination reached higher % values of sub-G1 cells in the H1975 cell line (~22%) when compared with A549 cells (~16%). Nevertheless, this effect was evident for both concentrations of MnTnHex in A549 cells (0.5 and 1 µM; Figure 5D), while for H1975 cells, significant results were only achieved with 1 µM (Figure 5G). 

For A549 cells, a mild G2/M arrest accompanied by a reduction in G0/G1 was observed when cisplatin and MnTnHex were added in combination, while the individual treatment with each of these compounds did not have a significant effect (Figure 5E,F). Overall, these results suggest that the observed reduction in cell viability present in the combinatory study mentioned in Section 3.3 and depicted in Figure 4 could be ascribed to the induction of cell death. 

### 3.5. MnTnHex Reduces Collective Cell Migration

To guarantee that the findings obtained with the wound-healing assay were due to an impairment in cell migration and not a consequence of reduced cell viability, it was necessary to first determine which non-toxic concentrations of MnTnHex and cisplatin should be used. For that, A549 cells were treated with two low concentrations of MnTnHex (1 and 5 µM) and cisplatin (0.25 and 0.5 µM) (Figure S1A), and H1975 cells were treated with MnTnHex (0.25 and 0.5 µM) and cisplatin (0.5 and 1 µM) (Figure S1B). All experiments were performed using a culture medium with 2% FBS, and cytotoxic effects were assessed using the MTS reduction assay. 

In A549 cells, both cisplatin concentrations displayed 100% cell viability. For MnTnHex, both concentrations tested caused only a minor decrease in cell viability. Therefore, we selected the representative non-toxic concentrations of 0.5 µM for cisplatin and 5 µM for MnTnHex. In H1975 cells, none of the two concentrations of cisplatin tested showed cytotoxic effects. For MnTnHex, the concentrations of 0.25 µM and 0.5 µM had a decrease in cell viability of less than 10%. Hence, we chose the non-toxic concentrations of 1 µM for cisplatin and 0.5 µM for MnTnHex.

**Figure 6:**
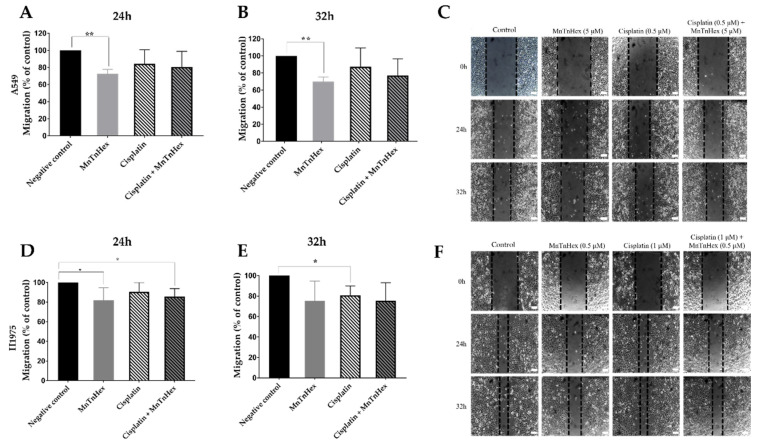
Effect of MnTnHex alone or combined with cisplatin on the collective migration of NSCLC cells. Cell migration was evaluated by wound-healing assay using MnTnHex (5 μM) and/or cisplatin (0.5 μM) in A549 cells at (**A**) 24 h and (**B**) 32 h. (**C**) Representative microscopy images of the wound-healing assay of A549 cells. Cell migration was also evaluated in H1975 cells at (**D**) 24 h and (**E**) 32 h using MnTnHex (0.5 μM) and/or cisplatin (1 μM). (**F**) Representative microscopy images of the wound-healing assay of H1975 cells. Values for the wound-healing assay represent mean ± SD (*n* = 3–4) and are expressed as percentages relative to control cells for each time point. The statistical analysis was performed for each time point, comparing each condition with the control cells; * *p* < 0.05, ** *p* < 0.01 (Student’s *t*-test). Scale bar = 100 μm.

After selecting the non-cytotoxic concentrations of both compounds, the migration capability of A549 and H1975 cells were assessed using the wound-healing assay (Figure 6), which evaluates the collective cell migration through a horizontal surface. In A549 cells, MnTnHex alone significantly reduced wound closure by approximately 30% (*p* < 0.01) at 24 h and 32 h (Figure 6A,B). When combined with cisplatin, this compound also tends to reduce cell migration. In H1975 cells, a significant reduction occurred at 24 h, either using MnTnHex alone or combined with cisplatin, of approximately 19% (*p* < 0.05) and 15% (*p* < 0.05), respectively (Figure 6D,E). A similar % of reduction was also observed at 32 h, although without statistical significance. 

### 3.6. MnTnHex Alone and/or Combined with Cisplatin Induced Alterations in the Metabolic Response of NSCLC Cells

**Figure 7:**
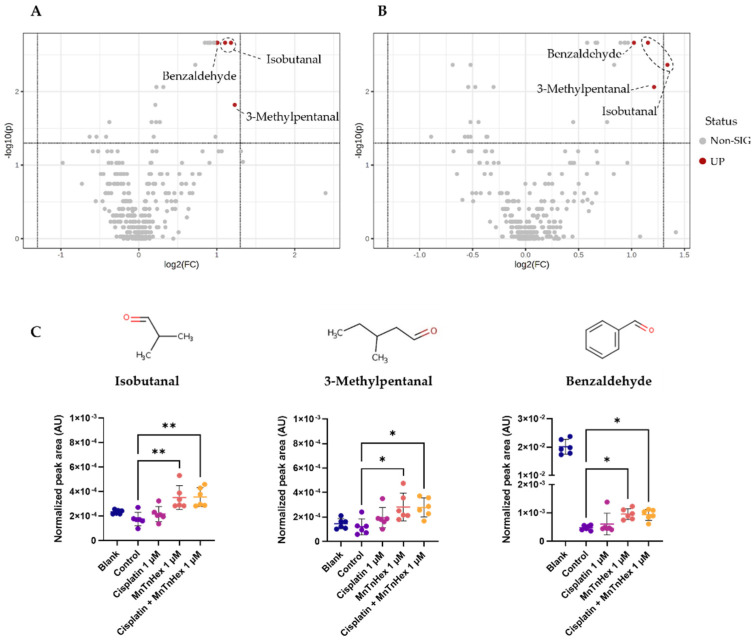
GC-MS-based metabolomics analysis of the extracellular media of H1975 cells exposed to MnTnHex and cisplatin, alone and combined. (**A**) Volcano plot of the GC-MS data of H1975 cells exposed to MnTnHex (1 μM) 72 h compared with controls. (**B**) Volcano plot of GC-MS data of H1975 cells co-exposed to Cisplatin (1 μM) and MnTnHex (1 μM) compared with controls, indicating the statistical significance (*p*-value) vs. the magnitude of change (fold change). Statistical significance was assessed using the Mann-Whitney test and *p*-values < 0.05 were considered significant. The grey dots indicate non-significant m/z-RT pairs, while the red dots indicate statistically significant m/z-RT pairs corresponding to isobutanal, 3-methylpentanal and benzaldehyde as marked. (**C**) Boxplots representing the normalized peak areas of the three significant VCCs. Statistical significance was assessed using the Kruskal-Wallis ANOVA test (* *p* < 0.05, ** *p* < 0.01).

First, the analytical reproducibility of the metabolomics experiment was confirmed by unsupervised analysis (PCA) of GC-MS data (Figure S3), including all samples under study and QCs, which revealed a well-defined QCs cluster. From a multivariate discriminant analysis (PLS-DA) perspective, no clear separation was found between the extracellular media of A549 or H1975 cells exposed to MnTnHex (0.5 and 1 μM) and cisplatin (1 μM) alone or in combination. Hence, altered levels of VCCs were investigated from a univariate analysis perspective using Volcano plots (Figure 7).

Among the 21 VCCs detected in extracellular media of both cell lines by our experimental approach (Table S1), statistically significant VCCs were only found in the extracellular medium of H1975 cells exposed to 1 μM of MnTnHex (Figure 7A) and 1 μM of MnTnHex combined with 1 μM cisplatin (Figure 7B) when compared with the control group. Cisplatin alone did not significantly increase levels of VCCs, possibly being an inferior external source of H_2_O_2_ relative to MnTnHex. Interestingly, significant alterations were observed for the same VCCs in both exposed groups (Figure 7C), namely isobutanal, 3-methylpentanal and benzaldehyde. The relative comparison with blanks (i.e., culture medium without cells), also represented in Figure 7C, allowed the interpretation of VCCs alterations in terms of release or uptake by cells. Thus, our findings unveiled that H1975 cells exposed to both conditions released significantly higher levels of isobutanal and 3-methylpentanal and uptook lower levels of benzaldehydebenzaldehyde than control cells. Although no statistically significant alterations were found for extracellular media of exposed A549 cells, the same trend was observed for isobutanal and benzaldehyde levels, showing a similar behavior for higher release of isobutanal and lower uptook of benzaldehyde after exposure to MnTnHex alone and combined with cisplatin (Figure S4). 

## 4. Discussion

The treatment of NSCLC requires improvement and additional drugs are necessary. Those could be used alone or in combination with chemotherapy to boost its efficacy and simultaneously overcome resistance and reduce its adverse side effects. A promising therapeutic strategy relies on using SODm, particularly MnPs, to increase ROS levels in cancer cells towards marked cytotoxic levels and induction of cell death. This strategy is based on the knowledge that malignant cells typically have higher levels of ROS and lower levels of some key antioxidant enzymes when compared to healthy cells [14,15,16,17].

Currently, one Mn porphyrin, the analog of MnTnHex, is being evaluated in Phase I/II cancer clinical trials (MnTnBuOE-2-PyP^5+^, BMX-001) to assess its efficacy when combined with chemotherapy or radiation. The advantages afforded by this redox-active drug in terms of healthy tissues are also one of the outcome measures evaluated (www.clinicaltrials.gov, accessed on 22 July 2022). The trials on BMX-001 are being conducted on glioma, head & neck cancer, anal cancer, and multiple brain metastases [25]. The in vitro and in vivo studies indicate that both Mn porphyrins are powerful anticancer drugs and protectors of normal tissues. The other analog, MnTE-2-PyP^5+^ (AEOL10113, BMX-010), is in Phase II clinical trial (NCT03381625) on atopic dermatitis and itch [25]. Our data on MnTnHex would not only contribute to the knowledge of the therapeutic potential of Mn porphyrins in other cancers but also to our understanding of their mechanism of action. 

MnPs have already been tested in several types of cancer in cellular and animal models. Yet, there is still a lack of information regarding the cytotoxic effects of these compounds in general and most so in lung cancer. Therefore, the goal of the present work was to assess the effect of MnTnHex on several aspects of lung cancer biology, including cell viability and motility. The innovative aspects of this study also include the in vitro evaluation of the combinatory effects of MnTnHex with cisplatin. Finally, we used exometabolome to address the oxidative stress-based lipid peroxidation induced predominantly by MnTnHex, via the analysis of the VCCs in a culture medium of NSCLC cells. 

The work presented here was carried out using two NSCLC cell lines. The A549 cell line is one of the most used cell lines, and as such, it allows us to compare our results to other reports. A549 cells display a K-RAS mutation [51] and are known for their sensitivity to cisplatin [52]. The H1975 cell line originated from a non-smoker female with lung adenocarcinoma and is considered highly invasive [53], with a mutation that confers resistance to EGFR inhibitors [54]. To better characterize both cell lines in terms of basal antioxidant gene expression, the relative expression of the genes (RT-qPCR) that code for the main H_2_O_2_ processing enzymes was carried out.

Our results showed that both NSCLC cell lines exhibited very low levels of CAT and relatively low levels of GPX1 and PRDX2. PRDX1 was highly expressed and is the predominant peroxiredoxin isoform in cells. According to the Human Protein Atlas (www.proteinatlas.org), comparing A549 cells with several other cell lines show that these cells rank along those with lower catalase levels. In addition, the levels of PRDX2 were undetected in A549 cells compared with other cell lines. Similar results in terms of gene expression were observed in studies with lung cancer. Some authors compared the antioxidant levels between tumor and tumor-free lung tissue. The lung tumor tissue showed lower levels of catalase expression than healthy tissue, while similar levels of GPX1 were detected in both cancerous and non-cancerous tissue. In addition, by performing the immunohistochemical localization of catalase, the results revealed lower or no expression of this enzyme in tumor cells [55]. Jiang et al. analyzed the expression of several PRDXs in multiple cell lines, and the absence of expression of PRDX2 was also found in A549 cells [56]. Overall, our analysis, along with the information reported elsewhere, pointed out the low gene expression level of these key enzymes, particularly catalase, in NSCLC cell lines, suggesting their propensity to changes in redox balance, thus anticipating the usefulness of the MnPs-based redox modulators, in the context of this type of cancer. Cancer cells use high levels of ROS to their own advantage for proliferation. Yet, the balance is delicate, and depending upon the levels of endogenous antioxidants, a tiny amount of external ROS may drive cancer cells to death. 

We first evaluated the cytotoxic impact of MnTnHex in both NSCLC cell lines using complementary methodologies. MnTnHex, as a single drug, exhibited a concentration-dependent decrease in terms of cell viability, revealing quite low IC_50_ values, in some cases reaching the sub-micromolar range. This fact, we believe, is of high relevance since it demonstrates the ability of this compound to act as a promising stand-alone drug in NSCLC. It should be emphasized that our previously published results with MnTnHex using cells of non-tumor origin clearly revealed opposite results. Indeed, this compound had already been assessed in V79 lung fibroblasts (MTT reduction assay, 24-h incubation) [33] as well as in renal cells (Vero cells) (CV assay, 48 h-incubation) [57]. In both cases, MnTnHex at concentrations up to 25 μM showed no evidence of cytotoxicity. Conversely, in our previous studies, MnTnHex already exhibited high cytotoxic effects in renal cancer cells (786-O) [29], which is in line with the herein observed results with NSCLC cells and demonstrates this important feature in different invasive cancer cells. While there are some experimental differences in the protocols used, it is clear that MnTnHex has differential effects in malignant vs. non-tumor cells. 

The gene expression data concerning catalase, and PRDX2, suggest that both cell lines are under high oxidative stress. Even without a cisplatin-derived source of H_2_O_2_, Mn porphyrins alone employ enough high levels of H_2_O_2_ to inflict oxidative damage to numerous enzymes, inactivating them and suppressing thus their activity [25,28]. Such results are supported by data showing that the IC_50_ values for MnTnHex in the present study are lower in both cell lines (between 0.7 µM and 2.1 µM depending on the type of assay and cell) than for cisplatin alone, indicating its potency in enhancing cellular oxidative stress beyond repair, and in turn death.

By combining MnTnHex with cisplatin, we further aimed to study a different aspect of the potential usefulness of this compound in NSCLC. That is its ability to increase the efficacy of chemotherapy treatment. Our group has been studying the sensitivity of cisplatin in different cell lines. Regarding A549 cells, these cells are more sensitive to cisplatin than H1975 cells having IC_50_ values up to 2.8 µM (Table S2). In the case of H1975 cells, the IC_50_ values for the CV and MTS assays were approximately 10 and 16 µM, respectively, as described in Manguinhas et al. [44]. Importantly, it should be mentioned that the IC_50_ values for MnTnHex in the present study are consistently lower than those observed for cisplatin, which fact is worthwhile to be emphasized. Regarding its role as a chemosensitizer, in the present study, when combined with cisplatin, MnTnHex was able to enhance the cytotoxic impact of this pivotal chemotherapeutic drug. Indeed, in both cell lines, MnTnHex increased the cytotoxicity of cisplatin, being much more effective in H1975 cells. This could be perceived as another beneficial aspect given the resistant pattern of these cells. We recently reported the same impact of MnTnBuOE-2-PyP^5+^ as a single drug in breast cancer cells, where even without carboplatin, the cytotoxicity of Mn porphyrin in its own right was massive [58]. We also saw a large effect of MnTnBuOE-2-PyP^5+^ in its own right as paclitaxel in a mouse ovarian xenograft study [59]. Regarding the differences observed in our study between the two NSCLC cell lines, several factors might be involved in their sensitivity to a given anticancer drug, including MnTnHex or cisplatin. The expression levels of the genes that code for H_2_O_2_-detoxifying enzymes may, at least partially, justify the differences observed between cell lines. Other reasons may involve differences between cell lines in terms of other enzymes involved in redox pathways, apoptosis, DNA repair, drug transport, and drug detoxification, among others.

Another aspect of this work was the characterization of the cell-cycle distribution of both cell lines treated with MnTnHex combined with cisplatin. For this, we selected a concentration of cisplatin that comprises low cytotoxicity (1 µM) and concentrations of MnTnHex that, albeit being cytotoxic, did not markedly decrease cell viability (Figure 2). This selection of concentrations was chosen with the purpose of better understanding the effective impact of a two-drug combination. The sub-G1 data revealed the cytotoxic potential of MnTnHex at 1 µM and recapitulated the impact of this MnP to boost the cytotoxicity of cisplatin in both cell lines. Regarding the cell cycle checkpoints, it is known that cisplatin can interfere at the G1/S and G2/M checkpoints [60]. The second checkpoint is generally more affected since cells trapped in G2/M can enter cell cycle arrest. At the concentration levels tested, we did not find these alterations. Nevertheless, when cisplatin was combined with MnTnHex, a mild but significant increase in the G2/M phase occurred, which may indicate that this combination promotes G2/M cell cycle arrest. However, this effect was only observed in A549 cells. A possible cause may be related to the long incubation period with the compounds. The G2/M arrest may happen early, and arrested cells will either be efficient at repairing damage or induce cell death; thus, at 72 h, these cells might have either died or progressed to G1/G0. 

MnTnHex induced a significant reduction of collective cell migration on both cell lines. Interestingly, there was a more significant reduction of migration in cells exposed to MnTnHex per se rather than when combined with cisplatin. Nonetheless, MnTnHex, either alone or combined, appears to induce a slightly higher reduction of migration when compared to cisplatin alone. The impact of MnTnHex on migration has already been studied in other types of cancer in combination with chemotherapy and radiotherapy. While in this study, MnTnHex was effective per se in reducing collective cell migration, the same was not observed in renal and breast cancer. In renal cancer, MnTnHex did not affect collective cell migration, although it significantly decreased chemotactic migration [29]. Flórido et al. thoroughly analyzed the effect of MnTnHex alone and combined it with dox in breast cancer cell lines (MCF7 and MDA-MB-231). This MnP per se did not alter the collective cell migration and chemotaxis. However, combined with dox, it significantly reduced cell motility and chemotactic migration on both cell lines [24]. When MnTnHex was combined with radiotherapy, it significantly reduced the migration and invasion of mouse mammary carcinoma 4T1 cells compared to control and radiation therapy alone. Similar to the results obtained in this work, MnTnHex as a single drug was the best in reducing migration [30].

Metabolomics is an emerging research field that has been used in recent years to gain further mechanistic data supporting the mode of action of drugs and other xenobiotics. In the area of SODm and other redox-active drugs, scarce information on this topic is available. Regarding the release/uptake of VCCs to/from a culture medium by NSCLC cells, we found higher levels of isobutanal and 3-methylpentanal. The increase in aldehyde levels is likely related to the MnTnHex/H_2_O_2_-driven catalysis of lipid peroxidation and/or oxidation of corresponding alcohols [61]. However, the results were only significant for H1975 cells. Of note, a similar trend in A549 cells was also observed. The treatment with higher concentrations of MnTnHex might result in more pronounced effects in both cell lines. Nevertheless, we aimed to evaluate this aspect of the metabolome at MnTnHex concentrations that were not markedly toxic, avoiding other potential interferences. Performing studies at lower levels of Mn porphyrin is also clinically more relevant; the BMX-001 analog is used in clinical trials at ~ 0.2 mg/kg (www.clinicaltrials.gov). 

Regarding the uptake of lower levels of benzaldehyde, other authors showed that this metabolite is uptaken by mammalian cells [62] and may be oxidized by aldehyde dehydrogenases (ALDHs) to the corresponding carboxylic acid [63]. These alterations appear to be a consequence of the mechanism of action of MnTnHex (Figure 6C) since a similar behavior was observed for H1975 cells exposed to MnTnHex alone and combined with cisplatin, whereas no significant changes were observed for cisplatin alone. Finally, it should be mentioned that the analysis of other metabolome alterations, for instance, in the endometabolome, may be important to better characterize the impact of MnTnHex in the context of NSCLC. These studies are currently undertaken using other methodologies, mostly nuclear magnetic resonance (NMR) spectroscopy, which may shed further light on this topic. 

## 5. Conclusions

The present study reveals that MnTnHex affects NSCLC cells in vitro at different levels. This redox-active drug, per se, markedly reduces the viability of NSCLC cells according to the endpoints explored, displaying very low IC_50_ values. It alters the cell cycle distribution and induces cell death. It further increases the cytotoxicity of cisplatin and most so in H1975 cells, suggesting its usefulness for combination therapy. Moreover, MnTnHex per se or combined with cisplatin reduces collective cell migration pointing out to be also effective in this important metastatic feature of malignancy. Finally, concerning the metabolomics, few VCCs associated with oxidative stress, likely resulting from MnTnHex/H_2_O_2_-induced catalysis of peroxidation, were identified in the culture medium of MnTnHex treated cells. Metabolomics deserves further exploration. Overall, this work suggests that MnTnHex should be considered a promising drug candidate for NSCLC. The research on MnTnHex, an analog of clinically-tested Mn porphyrin BMX-001, would also promote further development of Mn porphyrins into clinical trials on other types of tumors, NSCLC included.

## Figures and Tables

**Table 1:** Primers used for qRT-PCR characterization of NSCLC cells.

	Primer Sequence	Reference	Accession Number	
CAT	**F:** TGGAGCTGGTAACCCAGTAGG	[38]	NM_001752.4	
	**R:** CCTTTGCCTTGGAGTATTTGGTA			
GPX1	**F:** CGCTTCCAGACCATTGACATC	[39]	NM_000581.4	
	**R:** CGAGGTGGTATTTTCTGTAAGATCA			
PRDX1	**F:** CCACGGAGATCATTGCTTTCA	[40]	NM_001202431.2	
	**R:** AGGTGTATTGACCCATGCTAGAT			
PRDX2	**F:** CCTTCAAAGAGGTGAAGCTG	[41]	NM_005809.6	
	**R:** GTTGCTGAACGCGATGAT			

## Data Availability

Data is contained within the article or Supplementary Material.

## Supplementary Materials

The following supporting information can be downloaded at: https://www.mdpi.com/article/10.3390/antiox11112198/s1, Figure S1: Viability of NSCLC cells when exposed to low concentrations of cisplatin or MnTnHex in culture medium with 2% FBS and assessed by MTS assay; Figure S2: Representative HS-SPME-GC-MS chromatogram of the extracellular culture medium of the H1975 cells with identification of volatile carbonyl compounds (VCCs), as listed in the first column of the Table S1; Figure S3: Principal component analysis (PCA) scores plot of the HS-SPME-GC-MS chromatograms of extracellular media of all samples under study (H1975 and A549 cells exposed to MnTnHex alone and/or combined with cisplatin and controls, *n* = 71, green circles) and the quality control samples (QCs, *n* = 12, red circles) and Figure S4: GC-MS-based metabolomics analysis of the extracellular medium of A549 cells exposed to MnTnHex and cisplatin, alone and combined. Table S1: List of volatile carbonyl compounds (VCCs) identified in the extracellular culture medium of H1975 and A549 cells by HS-SPME-GC-MS. Table S2: IC_50_ values for MnTnHex and cisplatin in A549 and H1975 cells.
