# Supplementary material for: MnTnHex-2-PyP5+ Displays Anticancer Properties and Enhances Cisplatin Effects in Non-Small Cell Lung Cancer Cells
Source: Antioxidants (Basel). 2022 Nov 7;11(11):2198. doi: 10.3390/antiox11112198 (PMC9686800; doi:10.3390/antiox11112198)
Supplement: Supplementary file 1 [file antioxidants-11-02198-s001.zip › antioxidants-1945716-supplementary.pdf]

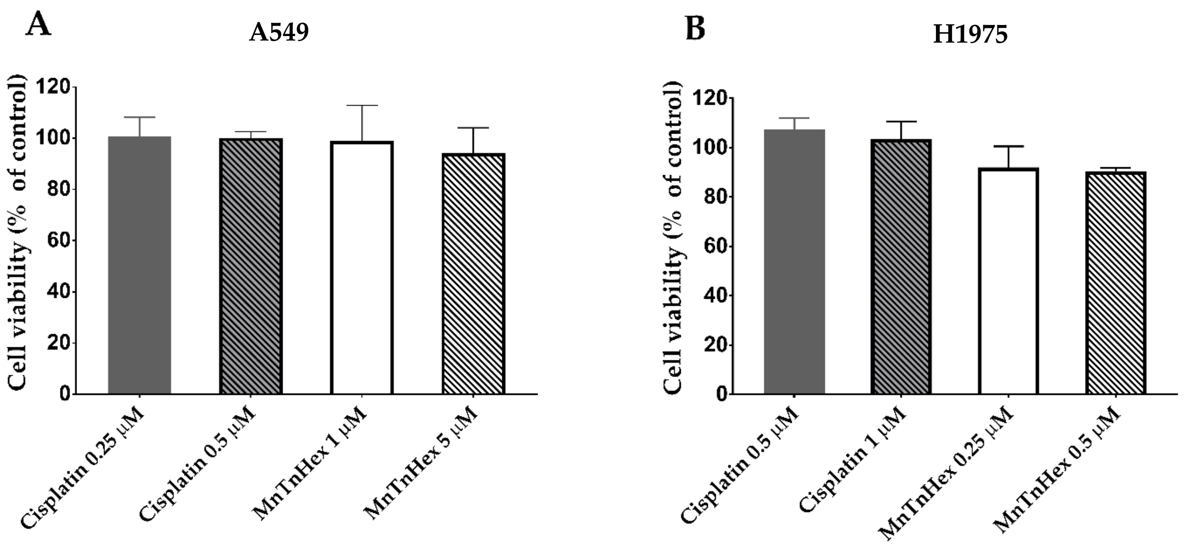

Figure S1. Viability of NSCLC cells when exposed to low concentrations of cisplatin or MnTnHex in culture medium with 2% FBS and assessed by MTS assay**.** Effect of cisplatin (0.25 and 0.5 µM) and MnTnHex (1 and 5 µM) on cell viability in A549 cells, in the presence of 2% FBS (**A**). Effect of cisplatin (0.5 and 1 µM) and MnTnHex (0.25 and 0.5 µM) on cell viability in H1975, in the presence of 2% FBS (**B**). Values represent mean ± SD (n=3) and are expressed as percentages relative to vehicle-treated control cells.

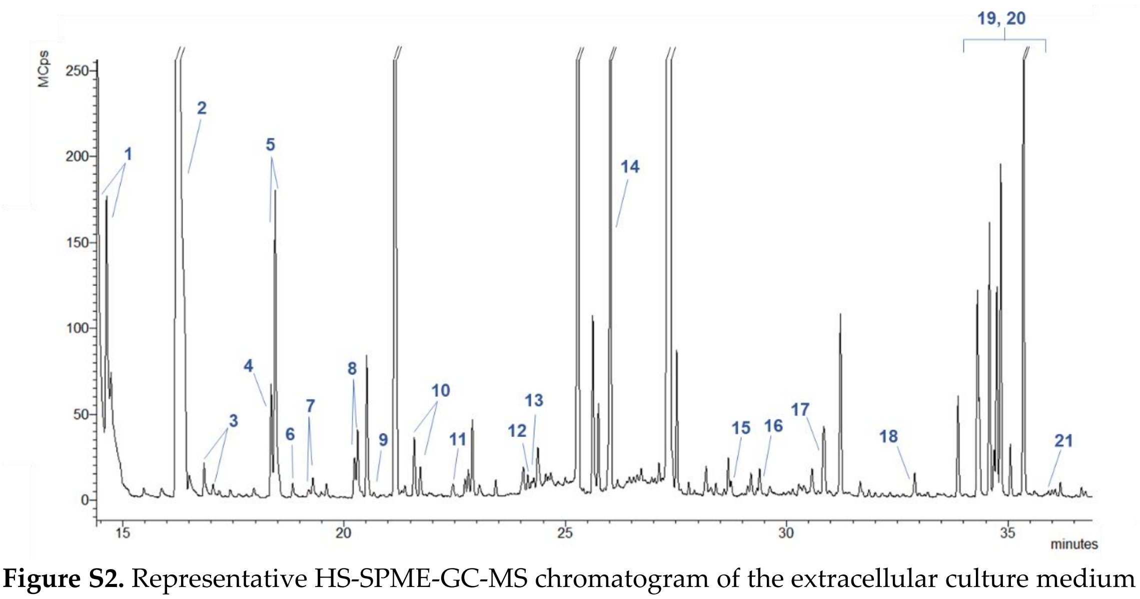

Figure S2. Representative HS-SPME-GC-MS chromatogram of the extracellular culture medium of the H1975 cells with identification of volatile carbonyl compounds (VCCs), as listed in the first column of the Table S1.

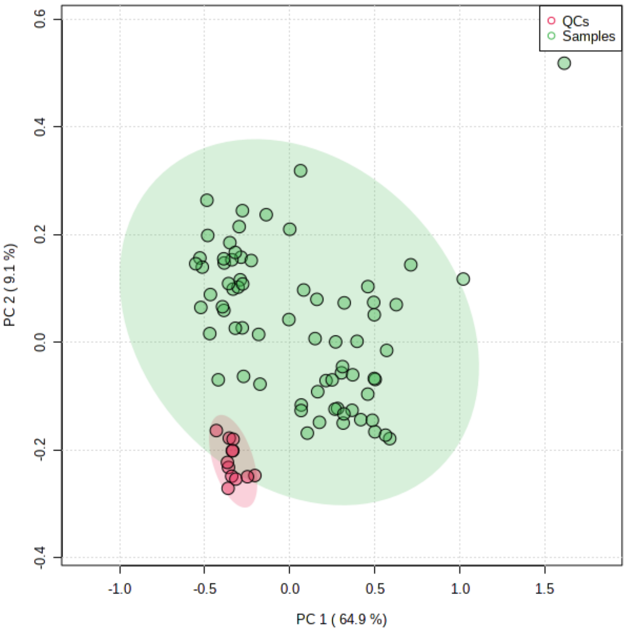

Figure S3. Principal component analysis (PCA) scores plot of the HS-SPME-GC-MS chromatograms of extracellular media of all samples under study (H1975 and A549 cells exposed to MnTnHex alone and/or combined with cisplatin and controls, *n*=71, green circles) and the quality control samples (QCs, *n*=12, red circles).

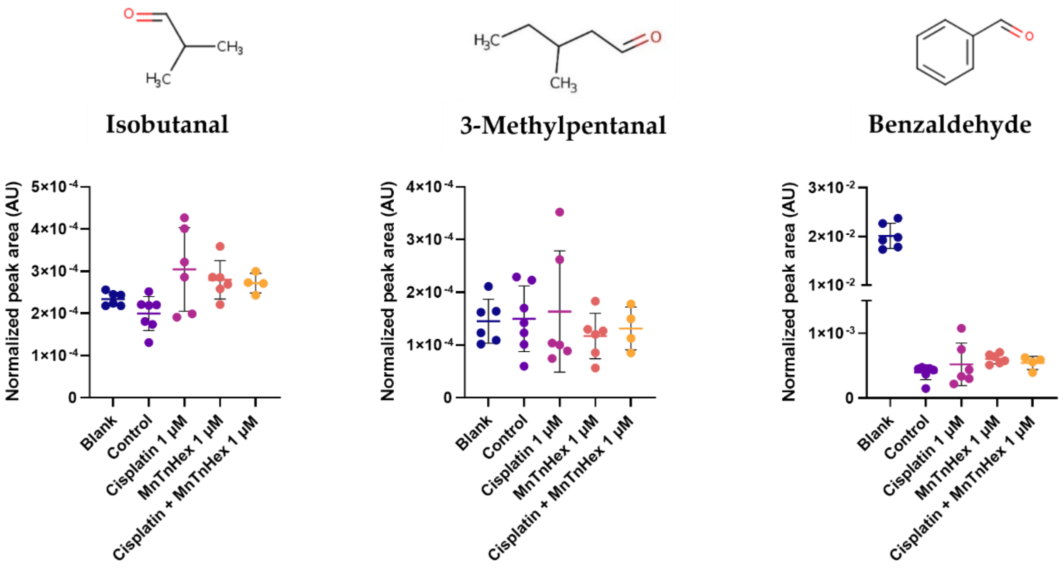

Figure S4. GC-MS-based metabolomics analysis of the extracellular medium of A549 cells exposed to MnTnHex and cisplatin, alone and combined. Boxplots representing the normalized peak areas of the three VCCs (isobutanal, 3-methylpentanal and benzaldehyde).

Table S1. List of volatile carbonyl compounds (VCCs) identified in the extracellular culture medium of H1975 and A549 cells by HS-SPME-GC-MS.

Nº	Compound	RT (min)	*m/z*	Rematch
1	acetaldehyde	14.43 / 14.64	117, 161, 209	928
2	acetone	16.23 / 16.41	161, 206, 253	927
3	propanal	16.84 / 17.04	161, 195, 236	903
4	isobutanal	17.96	195, 250	893
5	2-butenal	18.36 / 18.45	161, 195, 250	885
6	pentanal	19.30	161, 195, 239	860
7	3-methyl-2-butanone	19.61 / 19.75	100, 253, 281	793
8	2-pentanone	20.31 / 20.51	195, 236, 253	872
9	3-methylpentanal	20.39	161, 239	792
10	4-methyl-2-pentanone	21.37 / 21.88	236, 253, 295	794
11	2-hexanone	22.47 / 22.74	195, 253, 236	853
12	heptanal	24.15	239, 252	816
13	cyclopentanone	24.24	232, 279	721
14	cyclohexanone	26.02	195, 276, 293	856
15	octanal	28.68	207, 239	729
16	benzaldehyde	29.40	271, 301	827
17	nonanal	30.84	207, 239	851
18	decanal	32.89	207, 239	817
19	glyoxal	34.59 / 34.69 / 34.75	161, 252, 448	921
20	methylglyoxal	34.83 / 35.06 / 35.36	117, 265, 462	910
21	dimethylglyoxal	35.92	279, 476	792

Table S2. IC_{50} values for MnTnHex and cisplatin in A549 and H1975 cells.

Drug	Cell line	IC_{50} CV (μM)	IC_{50} MTS (μM)
MnTnHex	A549	0.9	2.1
	H1975	0.7	1.0
Cisplatin	A549	1.8	2.8
	H1975	9.6*	15.9*

*Data from Reference [44]; CV, Crystal Violet assay; MTS (3-(4,5-Dimethylthiazol-2-yl)-5-(3-carboxylmethoxyphenyl)-2-(4-sulfophenyl)-2H-tetrazolium).
